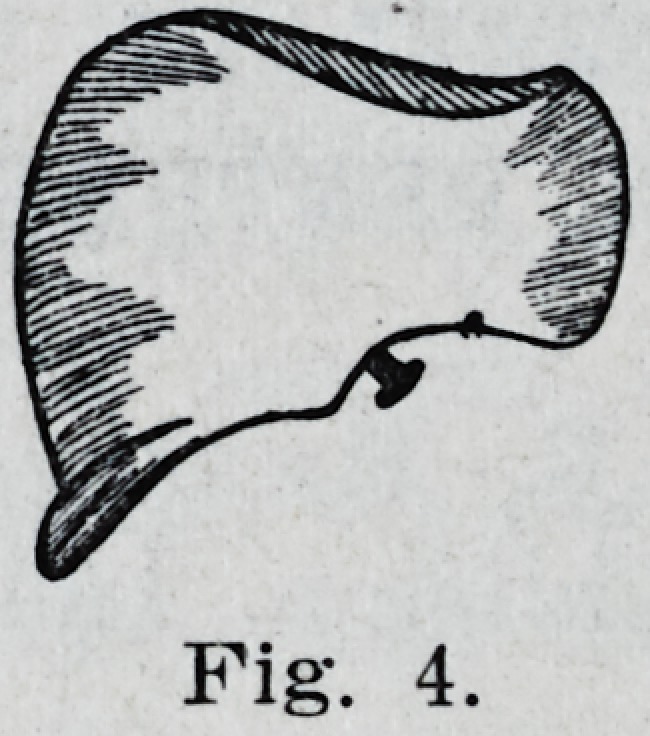# Grinding Porcelain Teeth for Full Dentures

**Published:** 1904-09

**Authors:** Frederick R. Henshaw

**Affiliations:** Middleton, Ind.


### GRINDING PORCELAIN TEETH FOR FULL DENTURES.

By Frederick R. Henshaw, D. D. S., Middleton, Ind.

(Written for *The American Journal of Dental Science.*)

How to make a plate that restores the features, the speech, the expression and the masticatory function of a patient, is a question that has tried the soul of many an earnest worker in our ranks.

If he were earnest enough in his efforts he probably solved the problem both to his own satisfaction and the comfort and delight of his patient; if he were not he may have produced one of those enormities that we see on every hand, collected his fee and then prayed that he might never see that particular patient again.

The fact remains undisputed that no operation in the field of Dentistry requires so much of painstaking effort as well as skill and ingenuity as the making of full dentures. One must have been a good student of the human face to look at a patient having an edentulous mouth and by the characteristics thereon written, select for that mouth of the correct shade, size and mold of teeth to restore it to a naturel appearance and condition.

The character of the teeth for the basal temperaments has been well defined but who, in America where there has been an indiscriminate mixture of races, ever saw an individual showing all the characteristics of any of the basal temperaments?

This matter of the proper selection of teeth at once becomes a serious one for the man who is anxious to do artistic work, and where the extraction of the teeth comes within his hands he would do well to make note at that time of the size, shape and color of the natural organs for future reference.

Seemingly, there has been a woeful lack of attention paid to plate work by the better men of the profession in late years that can only be accounted for by the difficulty that is almost universally experienced in obtaining adequate recompense for the time, labor and skill necessary to do this work as it should be done.

Much of this work is relegated to the dental laboratory and the man who stands responsible for it really has nothing to do with it.

One of the difficulties that has always stood in the way of making a perfect set of artificial teeth has been the failure on the part of the manufacturers to furnish teeth that can be readily adapted to the various cases.

One of the chief faults has been in the size of the bicuspids and molars. Few molds of the standard makes of teeth have bicuspids wide enough bucco-lingually nor molars broad enough mesio-distally. Neither is it possible for the dentist to mount the teeth in proper articulation with one another without first more or less extensively changing the shape of the occluding surfaces.

The writer described a method of grinding the occluding surfaces of the teeth in a paper published in the Dental Summary February 1904. Since then it has been found possible to make nearly as perfect an articulation in a much easier and more simple way, which will be here described.

In mounting the teeth the six anterior superior teeth should be mounted first and as the visible character of the work depends upon these teeth, great care should be exercised in mounting them, the incisal ends being ground to represent the necessary attrition that is nearly invariably present in the natural organs, and the contour so arranged as to give the most artistic and natural appearance obtainable.

The bicuspids should then be ground upon the occlusal ends. This is best done with a round faced, ½ inch, carborundum wheel in the engine. In the finished tooth, as it comes to us, the cusps are round and blunt and it is impossible to obtain more than a very imperfect contact with the opposing teeth.

By grinding the tooth on each side of the transverse ridge until it presents a concave surface on each side we obtain a result as in Fig. 1. By a little practice this can be done in a surprisingly short time and all of the upper bicuspids can be so treated at the same time.

Fig. 1.

Fig. 2.

In grinding the upper molars the occlusal surface is simply flattened from the lingual cusps toward the buccal, cutting into the buccal cusps about ½ their width and to a depth of the grooves as shown in Fig. 2. While this may take a little longer than for the bicuspids the results will more than compensate for the time. After all the superior teeth are ground to the desired shape and placed upon the model in correct position, we are ready for the lower.

Usually a very little grinding of the incisal ends of the six anterior teeth will suffice, merely sufficient to insure perfect contact in biting, the ends being beveled slightly inward.

The cuspids require the greatest amount of grinding, nearly always having to be shortened and beveled inward.

In grinding the bicuspids, the buccal cusps must be greatly shortened and the bucco-occlusal margin beveled toward the lingual to allow it to play directly against the concavity of the superior tooth. The two lateral sulci are ground to a concave that will permit them to come in contact with the similarly shaped surfaces in the lingual cusps of the superior teeth as in Fig. 3.

Fig. 3.

Each tooth must be ground and fitted into occlusion separately. By careful practice these teeth may be made to interdigitate so perfectly that there will be almost unbroken contact throughout their whole extent.

The lower molars are ground from the buccal margin toward the lingual, making the surface flat and cutting a groove across the lingual cusps as in Fig. 4.

Fig. 4.

When ground in this manner, ordinary porcelain teeth occlude so accurately that the question of utility is effectually disposed of, and, although there is considerable extra labor and time expended, the results will much more than compensate.

---

Principles of Cavity Preparation.—First establish cavity outlines; second, remove softened dentin; third, give cavity proper shape; fourth, trim and smooth enamel margins; fifth, the cavity toilet.—*Dental Pedagogics.*

## Figures and Tables

**Fig. 1. f1:**
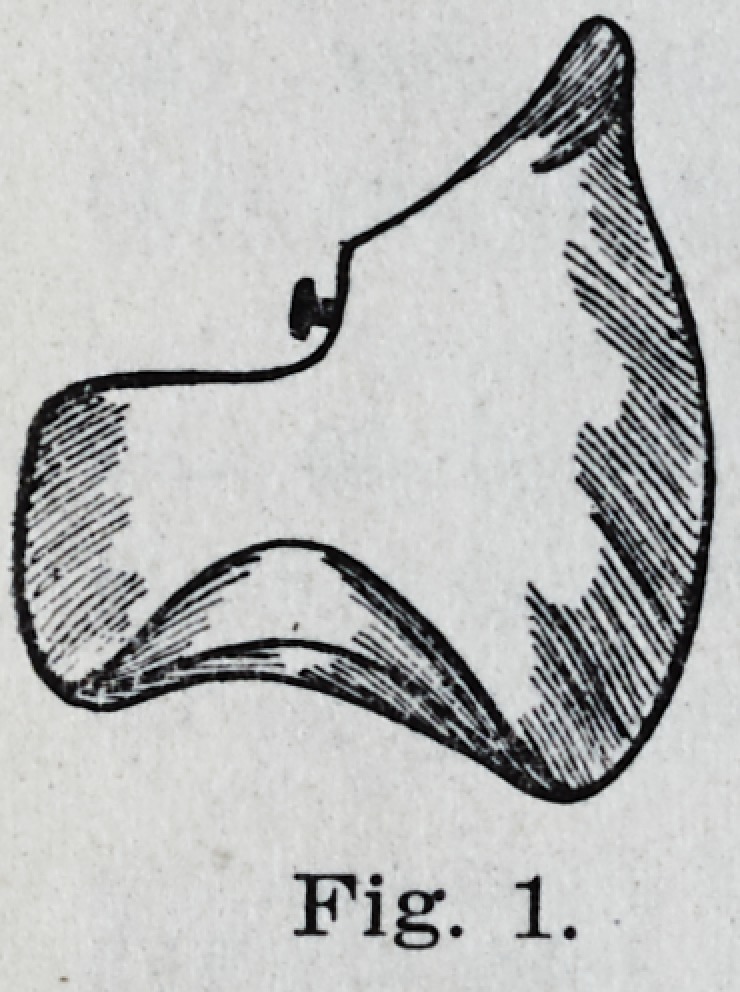


**Fig. 2. f2:**
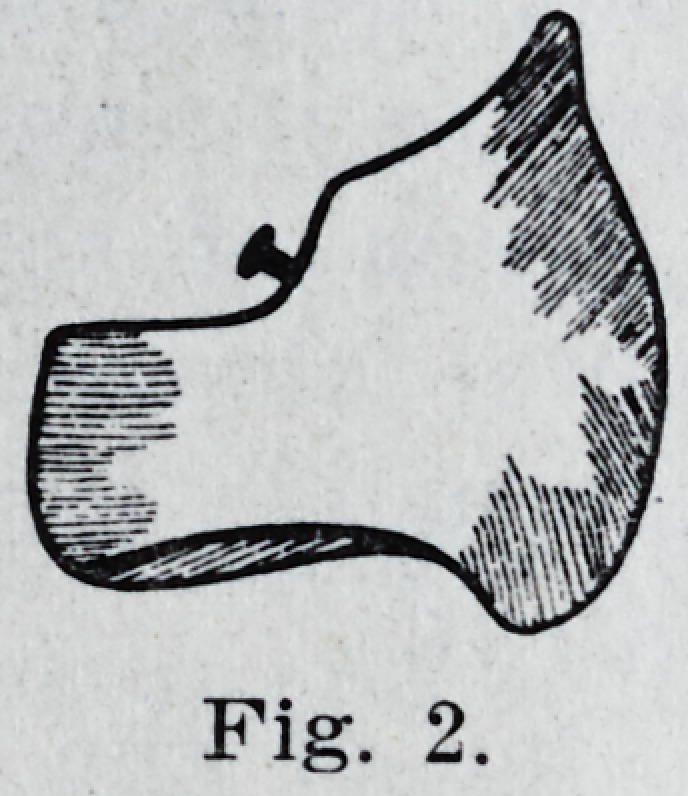


**Fig. 3. f3:**
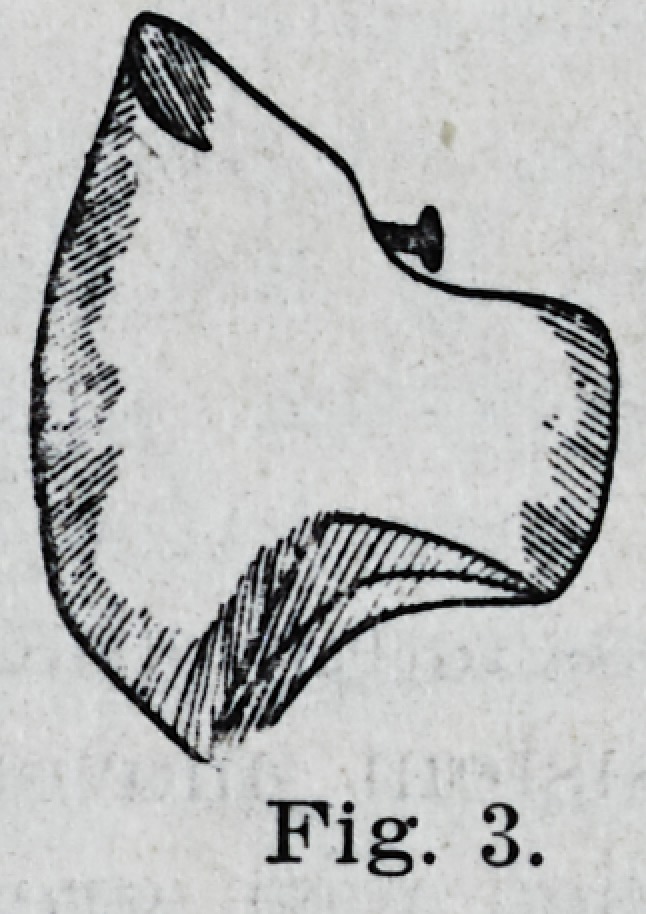


**Fig. 4. f4:**